# Cancer Therapy-Induced Cardiotoxicity: Results of the Analysis of the UK DEFINE Database

**DOI:** 10.3390/cancers17020311

**Published:** 2025-01-19

**Authors:** Stefanie Ho Yi Chan, Raymond W. Fitzpatrick, Deborah Layton, Sherael Webley, Sam Salek

**Affiliations:** 1 School of Life and Medical Sciences, University of Hertfordshire, Hatfield AL10 9AB, UK; 2 Department of Pharmaceutics, UCL School of Pharmacy, London WC1N 1AX, UK; 3 Centre for Medicines Optimisation, School of Allied Health Professionals and Pharmacy, Keele University, Newcastle ST5 5BG, UK; r.fitzpatrick@keele.ac.uk; 4 PEPI Consultancy Limited, Southampton SO53 1GR, UK

**Keywords:** non-small cell lung cancer, non-small cell lung cancer treatments, cardiotoxicity, cardiovascular adverse events, pharmacoepidemiology

## Abstract

Background: The accelerated development of novel cancer therapies necessitates a thorough understanding of the associated cardiotoxicity profiles, due to their significant implications for the long-term health and quality of life of cancer survivors. Objectives: The aim of this study was to determine the association between cardiotoxicity and non-small cell lung cancer (NSCLC) treatments using a hospital medicines usage database in England. Methods: An observational study based on a retrospective design using real-world data from the UK DEFINE database was performed. Monthly secondary data of 40 shortlisted drugs from April 2017 to July 2022 were extracted. Results: The cardiology drug that was associated with most oncology drugs was apixaban. Atezolizumab, bevacizumab, nintedanib, osimertinib, paclitaxel, pembrolizumab, gemcitabine and vincristine were all mostly associated with apixaban, which indicated association with atrial fibrillation. Afatinib, erlotinib and methotrexate were mostly associated with atenolol, hence suggesting the association with ischaemia or hypertension. Docetaxel and epirubicin were associated with verapamil, which indicated association with arrhythmia or hypertension. Conclusions: From the correlation and regression analyses, it can be concluded that hypertension was the most associated cardiovascular disease with the 20 shortlisted oncology drugs. The findings of this study have provided a better understanding of the association between each NSCLC–Cardio drug pair.

## Simple Summary

As cancer treatment advances with new therapies, understanding cardiovascular adverse events is crucial for improving the health and quality of life of cancer survivors. This study explored the association between heart-related complications and various cancer drugs by analysing real-world data from the UK DEFINE database. The study found that the blood thinner, apixaban, was commonly associated with several cancer drugs, thus suggesting the risk of atrial fibrillation, a type of irregular heartbeat. Other drugs like atenolol were connected to conditions like ischaemia (reduced blood flow) or high blood pressure. It also suggested that high blood pressure was the most common heart issue linked with these cancer treatments. By providing clearer insights into the cardiotoxic risks associated with specific cancer drugs, this research seeks to inform safer treatment choices, thereby enhancing patient care in oncology.

## 1. Introduction

In recent years, there has been a breakthrough in the development of novel targeted oncology drugs [1,2,3,4]. There was an increase of 22% oncology trials starting in 2022, compared to that of 2018 [5]. According to the Global Oncology Trends 2023, there was an average of 23 novel oncology therapeutic drugs launched annually from 2018–2022 and a total of 237 since 2003 [5]. Due to this continuous innovation and balanced by the rising adoption of biosimilars in major markets, the global spending on cancer medications increased to USD 196 billion in 2022 and is projected to reach USD 375 billion by 2027 [5].

With the rapid development of novel cancer treatments, an in-depth understanding of the associated cardiotoxicity profiles is of paramount importance due to its potential impact on cancer survivors’ long-term health and quality of life. Cardiotoxicity was first identified in 1967 in leukaemia patients treated with daunomycin, a type of anthracycline [6]. Subsequent reports in the early 1970s detailed an increasing incidence of anthracycline-induced cardiotoxicity. Anthracyclines induce cardiotoxicity through multiple mechanisms, including abnormal autophagy, dysregulated homeostasis of calcium ions, mitochondrial dysfunction and oxidative stress [7,8]. In the following decades, a broader spectrum of cardiotoxic effects attributed to various oncological agents has been observed [9,10]. It is crucial to identify and manage cardiotoxicity early on, so to allow for timely intervention and minimising long-term cardiac complications. The role of biomarkers is important in detecting early stages of cardiotoxicity, so to prevent damages caused by anticancer drugs. Abnormal expression or change in levels of biomarkers are indicators for screening and assessing the risk factors for cardiotoxicity complications. Current biomarkers include left ventricular ejection fraction (LVEF), troponin, brain natriuretic peptide (BNP), interleukin-6 (IL-6) and plasma myeloperoxidase [11,12].

According to the GLOBOCAN 2022 database released by the International Agency for Research on Cancer (IARC), it was estimated that there were 20 million new cancer cases and 9.7 million cancer deaths worldwide in 2022 [13]. Lung cancer was the most commonly diagnosed cancer worldwide with an estimated 2.48 million new cases, and also the most common cause of cancer death with an estimated 1.82 million deaths [13]. Non-small cell lung cancer (NSCLC) is the most common type of lung cancer, and its treatment involves various approaches including chemotherapies, targeted therapies and immunotherapies. Each of these treatments can potentially lead to cardiotoxicity, and thus impacting the heart via different mechanisms. For example, platinum-based treatments can cause oxidative stress and inflammation in cardiac cells, which lead to ischaemic complications, arrhythmias and hypertension. The vascular toxicity of these drugs can further contribute to thromboembolic events [14]. Studies suggested that angiogenesis inhibitors, i.e., bevacizumab, primarily increase the risk of cardiac ischaemia, hypertension and thromboembolic events, which then cause other cardiovascular adverse events such as myocardial infarction and heart failure [15]. Tyrosine kinase inhibitors (TKIs) specifically block tyrosine kinases, which are enzymes involved in the signalling pathways that regulate cell growth and survival. Despite their therapeutic benefits, TKIs can induce cardiotoxic effects that potentially limit their use. TKIs can interfere with mitochondrial function in cardiomyocytes, which lead to decreased adenosine triphosphate (ATP) production and increased oxidative stress. Mitochondria are crucial for energy production in heart cells, and their impairment can lead to energy deficits in the heart, and consequently contributing to reduced myocardial contractility and heart failure. In addition, TKIs can trigger apoptosis in cardiac cells directly by activating pro-apoptotic pathways or indirectly through increased oxidative stress and mitochondrial dysfunction. This loss of cardiomyocytes diminishes the heart’s ability to function efficiently and maintain its structural integrity [16,17].

Real-world data (RWD) is increasingly recognised as a crucial asset in regulatory science, hence transforming the landscape of healthcare regulation. This refers to the data collected outside the confines of randomised controlled trials (RCTs), offering insights into patient outcomes, medication adherence and treatment variability in real-world settings. As a result, it provides a more comprehensive understanding of a drug’s effectiveness and safety [18,19]. It can support healthcare treatment decisions and has increasingly been used to support decision making by regulatory bodies, such as the United States Food and Drug Administration (FDA), the European Medicines Agency (EMA) and the Medicines and Healthcare products Regulatory Agency (MHRA) [20].

Therefore, the aim of this study was to determine the association between cardiotoxicity and NSCLC treatments, as well as to describe the overall utilisation of the shortlisted drugs (20 oncology drugs and 20 cardiology drugs), using real-world data from the United Kingdom (UK) DEFINE database.

## 2. Methods

This study was a retrospective analysis of UK secondary care utilisation of shortlisted drugs in oncology and cardiology specialities using the DEFINE database in England. The DEFINE software (available from: https://rxinfo.thirdparty.nhs.uk [accessed on 26 August 2022]) is a National Health Service (NHS) prescribing database of medicines usage, covering 100% of NHS Secondary Care Trusts in England, as well as specialist centres and mental health trusts throughout the UK. It was developed by the software company Rx-Info (Exeter, UK) in conjunction with the West Midlands Regional Pharmaceutical Officer and the Chief Pharmacist of Royal Wolverhampton NHS Trust.

For this research, forty drugs were shortlisted—twenty cardiology and twenty oncology drugs. Twenty cardiology drugs were shortlisted following the outcomes of a previous systematic review by Chan et al., which identified the cardiotoxicities most commonly associated with NSCLC [21]. These 20 cardiology shortlisted drugs are used to treat the eight most frequently observed cardiotoxicities identified in that systematic review—arrhythmia, arterial/venous thromboembolic event, atrial fibrillation, cardiac failure/arrest, hypertension, ischaemia, myocardial infarction and tachycardia. The 20 shortlisted cardiology drugs were as follows: adrenaline/epinephrine, alteplase, amiodarone, amlodipine, anistreplase, apixaban, atenolol, bisoprolol, candesartan, diltiazem, doxazosin, lidocaine, lisinopril, losartan, ramipril, reteplase, rivaroxaban, streptokinase, tenecteplase and verapamil. Anticancer treatments were quantitatively ranked from most to least frequently used in patients with lung cancer (ICD-10 code: C34), based on data obtained from the Hospital Treatment Insights (HTI) database, from 1 January 2010 to 31 January 2020. Among the top 24 drugs listed in Table 1, etoposide and topotecan, which were solely used for small cell lung cancer (SCLC), were omitted from consideration as our study was focused on NSCLC. Celecoxib, a non-steroidal anti-inflammatory drug (NSAID) that selectively inhibits the cyclooxygenase-2 (COX-2) enzyme to minimise inflammation-related gastrointestinal toxicity [22]. Despite being investigated for its potential to boost the efficacy of chemotherapy and radiation therapy by increasing cancer cell susceptibility [23], celecoxib was excluded from the analysis. This exclusion was due to its broad application in non-cancerous conditions, such as osteoarthritis. Irinotecan, predominantly used for colorectal cancer and occasionally in off-label capacities for other cancers including NSCLC [24,25,26,27,28,29], was similarly excluded from the list. Following these exclusions, 20 NSCLC drugs were shortlisted for further investigation:

- Chemotherapy: carboplatin, cisplatin, cyclophosphamide, docetaxel, doxorubicin, epirubicin, gemcitabine, methotrexate, paclitaxel, pemetrexed, vincristine, vinorelbine;
- Targeted therapy: afatinib, bevacizumab, erlotinib, gefitinib, nintedanib, osimertinib;
- Immunotherapy: atezolizumab, pembrolizumab.

**Table 1:** Top 24 most commonly used lung cancer treatments (based on the number of patients with a C34 diagnosis and drug use recorded in the HTI database from 1 January 2010 to 31 January 2020).

NSCLC Drugs	Number of Patients with C34 Diagnosis and Drug Use
Carboplatin	20991
Cisplatin	8308
Pemetrexed	7839
Etoposide	7199
Gemcitabine	7015
Vinorelbine	6336
Pembrolizumab	3332
Docetaxel	2403
Erlotinib	2271
Cyclophosphamide	1657
Methotrexate	1359
Doxorubicin	1212
Vincristine	1120
Paclitaxel	1110
Atezolizumab	995
Gefitinib	752
Nintedanib	574
Afatinib	570
Celecoxib	488
Epirubicin	401
Topotecan	367
Osimertinib	345
Irinotecan	329
Bevacizumab	310

Monthly secondary care data from April 2017 to July 2022 were extracted from the DEFINE software. Data extracted were at gross national level or regional level, not at institutional or patient level. All statistical analyses were carried out through Microsoft Excel and/or R (version 4.3.3). All data were summarised to 3 decimal places. Descriptive analyses were conducted using number and percent within each category with 95% confidence intervals (whenever appropriate) for categorical variables, and mean (standard deviation [SD]), median (Q1, Q3), and minimum and maximum for continuous variables.

The counts and proportions of usage of cancer drugs and cardiology drugs were summarised by region and by national level. The association between the usage of the cancer drugs and cardiology drugs was explored by the Pearson’s correlation test (Equation (1)),

(1) $$r=\frac{\sum \left(x_{i}-\bar{x}\right)(y_{i}-\bar{y})}{\sqrt{\sum {\left(x_{i}-\bar{x}\right)}^{2}{\sum (y_{i}-\bar{y})}^{2}}}$$

where *r* is the Pearson correlation coefficient, $x_{i}$ represents the values of the *x*-variable in the sample and $y_{i}$ represents the values of the *y*-variable in the sample. The symbols $\bar{x}$ and $\bar{y}$ represent the means of the values of the *x*- and *y*-variables respectively.

Linear regression analysis was used to predict the value of a variable based on the value of another variable. The variable to be predicted is the dependent variable (*y*), while the variable that is being used to predict the other variable’s value is the independent variable (*x*). The aim of linear regression was to find the best-fitting straight line through the points of data. The equation for a simple linear regression model, which predicts a dependent variable *y* based on a single independent variable *x*, is given by

*y* = *β*_0_ + *β*_1_*x* + *ε* (2)

where *y* is the dependent variable to be predicted, *x* is the independent variable used to make predictions, *β*_0_ is the *y*-intercept of the regression line (representing the predicted value of *y* when *x* is 0), *β*_1_ is the slope of the regression line (representing the change in *y* for a one-unit change in *x*) and *ε* is the error term (representing the difference between the observed values and the values predicted by the model).

## 3. Results

**Figure 1:**
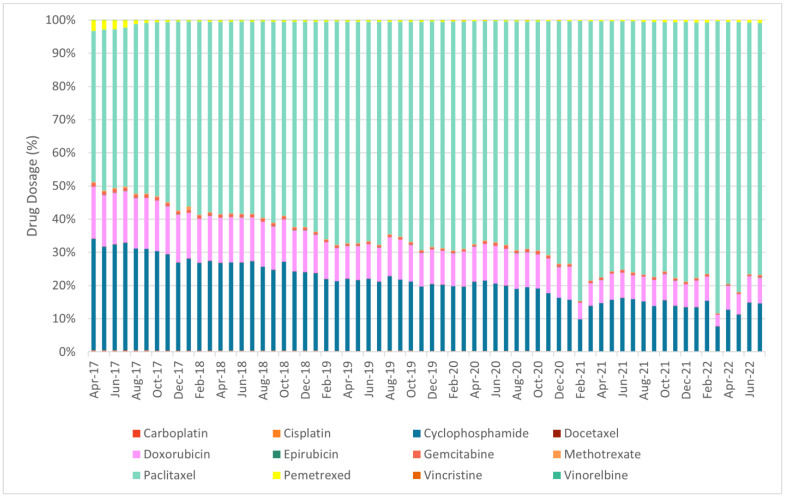
Distribution of utilisation of the shortlisted chemotherapies between April 2017 and July 2022 (at national level).

**Figure 2:**
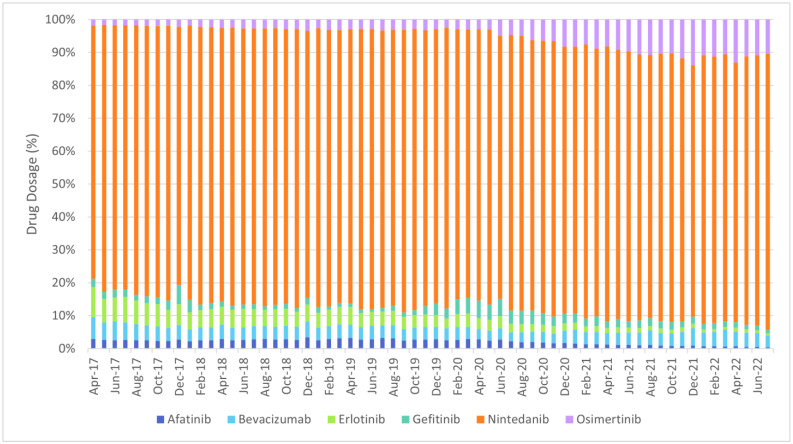
Distribution of utilisation of the shortlisted targeted therapies between April 2017 and July 2022 (at national level).

**Figure 3:**
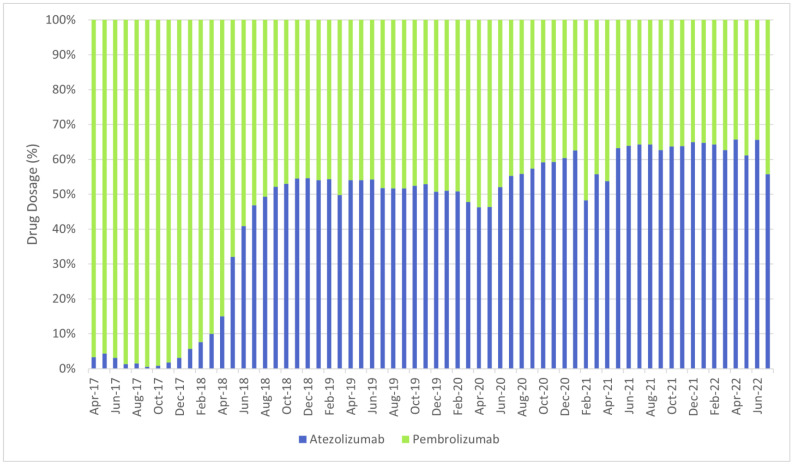
Distribution of utilisation of the shortlisted immunotherapies between April 2017 and July 2022 (at national level).

The defined daily dose (DDD), which is defined by the World Health Organization (WHO) as the mean maintenance daily dose of a medicine for its principal indication in adults, was meant to be used as the volume comparator. However, due to the nature of oncology treatments, i.e., the dosage of treatment for each patient can be different, the DDD is not available for oncology drugs. In addition, the unit of each drug is also different, hence the usage among each oncology drug can only be compared by trends instead of quantity. Figure 1, Figure 2 and Figure 3 show the overview of the distribution of utilisation of the shortlisted chemotherapies, targeted therapies and immunotherapies between April 2017 and July 2022 at national level.

The usage of atezolizumab increased from only 8.40 g in total at national level in April 2017 to 2832.00 g in total at national level in July 2022, with March 2022 recording the highest usage of atezolizumab (3024.11 g). The usage of osimertinib increased from 205.52 g in total at national level in April 2017 to 3320.48 g in total at national level in July 2022, with May 2022 recording the highest usage of osimertinib (3439.40 g).

It was discovered that London consistently ranked among the top four regions with the highest reported usage of all shortlisted oncology drugs between April 2017 and July 2022. This was possibly due to the presence of several cancer centres and hospitals in London, such as The Royal Marsden Hospital, University College Hospital Macmillan Cancer Centre and Nuffield Health Cancer Centre London (CCL).

**Figure 4:**
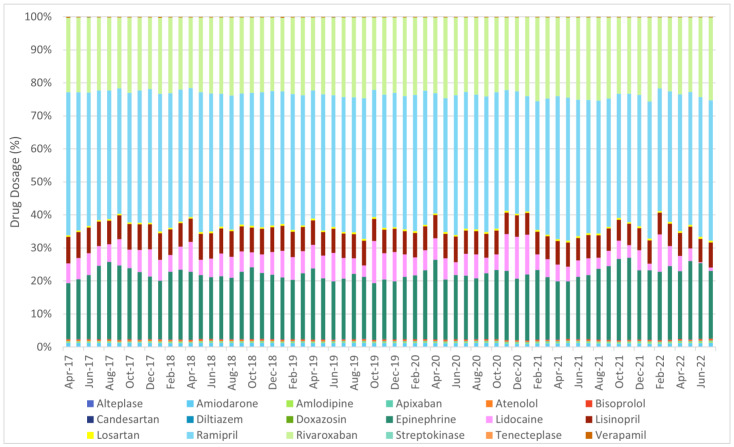
Distribution of utilisation of the 20 shortlisted cardiology drugs between April 2017 and July 2022 (at national level).

Among the 20 shortlisted cardiology drugs, it was discovered during the data extraction phase that there were no available data for anistreplase or reteplase. Figure 4 shows the overview of monthly utilisation of the 20 shortlisted cardiology drugs between April 2017 and July 2022 at national level.

A heat map showing the correlation between 20 shortlisted cardiology drugs and 20 shortlisted cancer treatments is presented in Table 2. The sign of the correlation coefficient (positive or negative) indicates the direction of the relationship between the independent and dependent variables. A positive coefficient means that as the independent variable increases, the dependent variable also increases, and vice versa for a negative coefficient. The closer the correlation coefficient is to +1, the stronger the positive correlation, whereas the closer it is to −1, the stronger the negative correlation. From the correlation analysis, it was demonstrated that the two drugs that were of the highest positive correlation were atezolizumab and apixaban (0.955), whereas erlotinib and apixaban showed the lowest negative correlation (−0.896).

Table 3 presents a summary linking each oncology drug with its most associated cardiology drug. From the cardiology drugs listed, the cardiovascular disease(s) associated with each oncology drug was deduced. The cardiology drug that was associated with the greatest number of oncology drugs was apixaban. Atezolizumab, bevacizumab, nintedanib, osimertinib, paclitaxel, pembrolizumab, gemcitabine and vincristine were all mostly associated with apixaban, which indicated association with atrial fibrillation. Afatinib, erlotinib and methotrexate were mostly associated with atenolol, hence suggesting the association with ischaemia or hypertension. Docetaxel and epirubicin were associated with verapamil, which indicated association with arrhythmia or hypertension. These drugs—amlodipine, atenolol, bisoprolol, candesartan, diltiazem, doxazosin, lisinopril, losartan, ramipril, and verapamil—are used to treat hypertension. Among the 20 shortlisted oncology drugs, 10 of them were most associated with one of the above-mentioned drugs, hence suggesting hypertension was the most common cardiovascular disease to be associated with the selected oncology drugs. Pearson correlation coefficients within each NSCLC drug group as well as the cardiotoxicity drugs group were calculated. There was evidence of strong correlations between these groups (Tables S1–S5).

**Figure 5:**
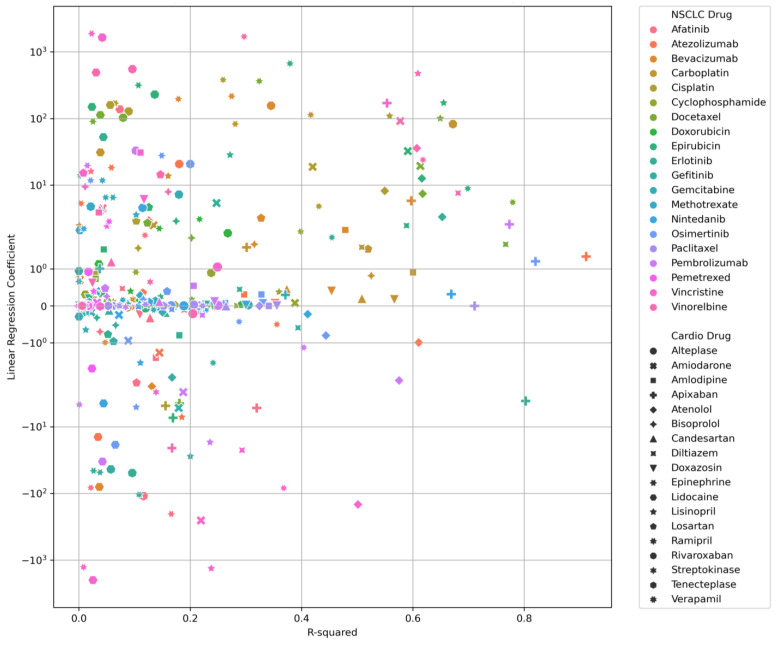
An overview of the predictive power of the regression models and the magnitude of the effect for each NSCLC–Cardio drug pair.

Figure 5 shows a scatter plot of linear regression coefficient versus R^2^ value for NSCLC–Cardio drug pairs. This plot provides insights into the relationship between the predictive power of the regression models (as indicated by the R^2^ value) and the magnitude of the effect (as indicated by the regression coefficient) for each NSCLC–Cardio drug pair. The colour of the points represents the NSCLC drugs while the shape of the points represents the cardiology drugs, and thus each point represents one NSCLC–Cardio drug pair. A high regression coefficient indicates a strong effect of the independent variable on the dependent variable. For each unit increase in the independent variable, the dependent variable increases (or decreases, if the coefficient is negative) by a large amount. This suggests a strong relationship between the two variables. A low regression coefficient suggests a weaker effect of the independent variable on the dependent variable. For each unit increase in the independent variable, the dependent variable changes by a small amount. This indicates a weaker relationship between the two variables. R^2^ values close to 1 indicate that the regression predictions perfectly fit the data.

There were seven drug–drug pairs with an R^2^ value of above 0.7, including (from highest) atezolizumab–apixaban, osimertinib–apixaban, erlotinib–apixaban, docetaxel–verapamil, pembrolizumab–apixaban, docetaxel–diltiazem and paclitaxel–apixaban. R^2^ values of 0.7 or higher are often considered strong, indicating that the model explains a substantial portion of the variance in the dependent variable. This indicated the possible association of atrial fibrillation with atezolizumab, osimertinib, erlotinib, pembrolizumab and paclitaxel, and hypertension with docetaxel. There were a further 29 drug–drug pairs of R^2^ values between 0.5 and 0.7, which might suggest a moderate relationship. The remaining drug–drug pairs were all of R^2^ values below 0.5, which might indicate a weak relationship, where the model does not explain much of the variance. The top right quadrant of the figure presents drug–drug pairs exhibiting positive correlations, whereas the bottom right quadrant showcases drug–drug pairs demonstrating negative correlations.

Table 4 shows the values of regression analysis for each drug–drug pair. For linear regression analysis, the following equation is applicable for all drug–drug pairs.

Cardiology Drug Usage = NSCLC Drug Usage × Coefficient + Intercept(3)

## 4. Discussion

According to the drug utilisation results, there was a dip across all NSCLC treatments in April 2020. This can be possibly related to the lockdown during the coronavirus disease 2019 (COVID-19) pandemic, as all but the most urgent of non-COVID care had to be cancelled, including many cancer treatments due to a lack of system capacity [30]. Moreover, it can be associated with the decreased number of cancer diagnoses during that time. According to the NHS, there was a notable 12% decline in the number of all cancer diagnoses in England in 2020, decreasing from 327,174 new cases in 2019 to 288,753. This marked a deviation from the typical small annual increases observed until 2019. In 2021, the total number of cancers diagnosed in England was back to 329,665. This represented a return to the gradual upward trend in annual cancer diagnoses that was interrupted by the pandemic in 2020 [31].

Atezolizumab works by binding to programmed death-ligand 1 (PD-L1), a protein found on the surface of cancer cells and certain immune cells. PD-L1 binds to programmed death-1 (PD-1) receptors on immune cells, which inhibits their ability to attack cancer cells. By blocking the PD-L1/PD-1 interaction, atezolizumab helps to unleash the immune system, allowing it to mount a more effective anticancer response [32]. Atezolizumab received its first FDA approval in May 2016. The initial approval was for the treatment of locally advanced or metastatic urothelial carcinoma, a type of bladder cancer, in patients who experienced disease progression during or following platinum-containing chemotherapy or within 12 months of neoadjuvant or adjuvant platinum-containing chemotherapy. In October 2016, the FDA approved atezolizumab for the treatment of metastatic NSCLC that progresses despite platinum chemotherapy. Atezolizumab has now received additional FDA approvals for various indications, including SCLC, triple-negative breast cancer (TNBC) and hepatocellular carcinoma (HCC).

The National Institute for Health and Care Excellence (NICE), which provides guidance on healthcare interventions in England, issued its positive recommendation for atezolizumab in August 2017. This recommendation made atezolizumab available through the NHS for the treatment of advanced urothelial carcinoma in patients who were not eligible for cisplatin-containing chemotherapy or had experienced disease progression within a year of receiving platinum-based chemotherapy. This explained why only since March 2018, atezolizumab was made available across the whole nation. Prior to that, only the North West region recorded usage of atezolizumab from April 2017. The South Central region recorded usage of atezolizumab from April 2017 to July 2017, and then again from January 2018 onwards. London and Yorkshire and the Humber started recording usage of this drug from November 2017. Since then, additional approvals and recommendations for atezolizumab have been granted by NICE for other cancer types, including NSCLC and TNBC. As of January 2022, atezolizumab was the first immunotherapy approved by the NHS for patients with early-stage NSCLC whose tumours express the PD-L1 mutation, and who have undergone surgery and chemotherapy. These patients are at risk of recurring cancer. England is currently the second country in Europe to make this cutting-edge treatment available. This explained the increasing usage of atezolizumab from only 8.40 g in total at national level in April 2017 to 2832.00 g in total at national level in July 2022.

Osimertinib is a third-generation epidermal growth factor receptor (EGFR) TKI. It selectively targets and irreversibly binds to mutated forms of the EGFR protein, including the most common EGFR mutations (EGFR exon 19 deletions or L858R substitution). By inhibiting the activity of mutated EGFR, osimertinib blocks the signalling pathways that promote cancer cell growth, division and survival [33]. Osimertinib received its first FDA approval in November 2015. The initial approval was for the treatment of patients with metastatic NSCLC whose tumours have specific EGFR mutations, specifically T790M mutations, and whose disease has progressed on or after EGFR TKI therapy. Since its initial approval, osimertinib has received additional FDA approvals for expanded indications, including first-line treatment of metastatic NSCLC with EGFR exon 19 deletions or exon 21 L858R mutations. NICE issued its positive recommendation for osimertinib in April 2018. This recommendation made osimertinib available through the NHS as a treatment option for advanced NSCLC with specific EGFR mutations in patients who have acquired resistance to previous EGFR TKI therapy. As of May 2021, osimertinib, which is believed to halve the risk of lung cancer patients suffering a return of the disease after undergoing treatment, was rolled out by NHS England. Although osimertinib was already available across the nation in April 2017, the positive recommendation by NICE in 2018 and official rolling out by the NHS in 2021 had contributed to an increase in the use of osimertinib from 205.52 g in total at national level in April 2017 to 3320.48 g in total at national level in July 2022, with May 2022 recording the highest usage of osimertinib (3439.40 g).

Atezolizumab, bevacizumab, nintedanib, osimertinib, paclitaxel, pembrolizumab and vincristine were all mostly associated with apixaban. Apixaban is an anticoagulant used for blood clots, e.g., in deep vein thrombosis and atrial fibrillation. Apixaban offers a favourable safety profile compared to warfarin, with lower rates of major bleeding and intracranial haemorrhage [34]. It also avoids the need for regular international normalised ratio (INR) monitoring, reducing the inconvenience and associated costs for patients. However, it is also important to note that the continuous increase in drug dosage of apixaban could be due to the shift from the use of vitamin K antagonists (VKAs) to direct oral anticoagulants (DOACs), according to the current NICE guideline, supported by evidence from the 2018 European Heart Rhythm Association Practical Guide on ‘The use of non-vitamin K antagonist oral anticoagulants in patients with atrial fibrillation’ [35], the British Committee for Standards in Haematology (BCSH) Guidelines on ‘Oral anticoagulation with warfarin—fourth edition’ [36], the Scottish Intercollegiate Guidelines Network (SIGN) guideline ‘Antithrombotics: indications and management’ [37], and on information in manufacturers’ Summaries of Product Characteristics (SPCs) and the British National Formulary (BNF).

For atrial fibrillation, treatments focus on rate control, rhythm control and stroke prevention. Rate control drugs include beta-blockers and calcium channel blockers [38]. Rhythm control may involve antiarrhythmic drugs, e.g., amiodarone, sotalol, flecainide and verapamil. Stroke prevention is critical, with anticoagulants such as warfarin or DOACs like apixaban, dabigatran, edoxaban and rivaroxaban being common choices. The specific drugs and treatment approach depend on individual patient factors, including symptoms, underlying heart condition and risk for stroke [39]. Antihypertensives such as amlodipine, atenolol, bisoprolol, candesartan, diltiazem, lisinopril, losartan and ramipril manage blood pressure and reduce atrial fibrillation complications. These drugs target different aspects of atrial fibrillation management, from preventing stroke to controlling heart rate and rhythm. In the UK, treatment is primarily guided by NICE, which emphasises stroke prevention using DOACs such as apixaban and rivaroxaban, alongside rate control with beta-blockers like atenolol, bisoprolol and sotalol or calcium channel blockers such as diltiazem and verapamil. The United States (US) follows the guidelines set by the American College of Cardiology (ACC), the American Heart Association (AHA), the American College of Clinical Pharmacy (ACCP) and the Heart Rhythm Society (HRS), collectively referred to as the ACC/AHA/ACCP/HRS guidelines. These guidelines prioritise DOACs for anticoagulation, with additional emphasis on individualised approaches to rate and rhythm control using medications like metoprolol and flecainide or procedural interventions such as catheter ablation. Meanwhile, the European Union (EU) adheres to the European Society of Cardiology (ESC) guidelines, which incorporate the AF-CARE framework, which stress the comprehensive management of comorbidities, risk factors and lifestyle modifications. While all regions prioritise DOACs for stroke prevention due to their safety and efficacy, procedural options like catheter ablation and rhythm control strategies are more frequently emphasised in the US and EU than in the UK.

As mentioned previously, afatinib, erlotinib and methotrexate were mostly associated with atenolol. Atenolol is a medication belonging to the class of drugs known as beta-blockers. It is primarily used to treat various cardiovascular conditions by blocking the effects of adrenaline on the beta receptors in the heart and blood vessels. Atenolol selectively blocks beta-1 adrenergic receptors in the heart, hence reducing the effects of adrenaline and other stress hormones, leading to a decrease in heart rate and cardiac contractility. This action helps lower blood pressure and reduce the workload on the heart, making it beneficial for managing certain cardiovascular conditions, such as angina, arrhythmias, hypertension and myocardial infarction. Atenolol is commonly prescribed to manage high blood pressure. By lowering heart rate and reducing the force of contraction, it helps to relax blood vessels and improve blood flow, thereby reducing blood pressure. It is also used in the treatment of stable angina by decreasing the heart’s oxygen demand and thus relieve symptoms and prevent angina attacks. Atenolol may also be prescribed to manage certain cardiac arrhythmias, such as supraventricular tachycardia and atrial fibrillation. It helps to stabilise heart rhythm by slowing the electrical impulses in the heart. It is sometimes used in the early phase of myocardial infarction (heart attack) as well to reduce the risk of recurrent events and improve survival [40].

The correlation matrix and regression analysis demonstrated the relationship between each NSCLC–Cardio drug pair. Based on guidelines of cardiovascular drugs used to treat cardiovascular diseases, it suggested that hypertension was the most associated cardiovascular disease with the 20 shortlisted oncology drugs. Certain oncology drugs, including targeted therapies and immunotherapies, can directly cause or contribute to hypertension as a side effect. These drugs may interfere with signalling pathways involved in blood vessel regulation, leading to increased blood pressure. Hypertension in cancer patients can have significant implications for cardiovascular health. Uncontrolled high blood pressure can increase the risk of cardiovascular events, such as heart attack, stroke or heart failure. Therefore, it is important to manage hypertension effectively to minimise these risks and ensure optimal cardiovascular health during cancer treatment.

The results derived from the correlation matrix and regression analysis can be used to indicate which drug–drug pair has the strongest association; however, it is crucial to note that these findings reflect correlation only and do not imply causation. This does not necessarily imply that one drug is used to treat a condition caused by the other or that the diseases treated by these drugs are directly related. Other factors may influence these correlations, and further investigation is necessary to determine any causal relationships.

A limitation of this study was that the data source only captured drug usage, so any suspected correlation could be purely coincidental as cardiology drugs can be used for various reasons and were not limited to use by NSCLC patients. Secondly, this study did not use individual patient-level data; therefore, it was not possible to evaluate whether the shortlisted cardiology and oncology drugs were administered to the same individuals. Thus, the correlations observed reflect patterns of drug use rather than direct evidence of increased cardiotoxicity caused by specific cancer drugs. Consequently, other unknown factors may also contribute to the observed associations. Another limitation was that this study time frame (~5 years) may be insufficient to observe long-term trends, particularly for chronic conditions or long-term adverse events that might emerge with prolonged drug use. Moreover, some of the traditional chemotherapy drugs are used to treat multiple cancers, and in this database, it was not possible to capture the usage of these drugs by NSCLC patients alone. As a result, describing the correlation as exclusively NSCLC-related might not fully reflect the complexity of the data. Additionally, it was not possible to divide all NSCLC drugs by the DDD to obtain actual patient numbers as each patient has varying treatment dosage and duration. Furthermore, cardiology drugs, such as apixaban, might be used to treat adverse events, e.g., acute thrombosis, rather than indicating associated cardiotoxicity from pembrolizumab. This limitation suggests the need for additional validation through screening assays and cheminformatic analysis to confirm the associations with drug–drug interactions, which is beyond the scope of this study and could be explored in future research.

## 5. Conclusions

The findings of this study have provided a better understanding of the association between each NSCLC–Cardio drug pair. The cardiology drug most frequently associated with oncology drugs was apixaban. Specifically, atezolizumab, bevacizumab, nintedanib, osimertinib, paclitaxel, pembrolizumab, gemcitabine and vincristine were predominantly linked to apixaban, suggesting a notable association with atrial fibrillation. In contrast, afatinib, erlotinib and methotrexate were primarily associated with atenolol, indicating a connection with ischaemia or hypertension. Additionally, docetaxel and epirubicin were correlated with verapamil, which is indicative of an association with arrhythmia or hypertension. The correlation matrix revealed that hypertension was the cardiovascular condition most frequently associated with the 20 shortlisted oncology drugs, underscoring its prevalence in patients undergoing cancer treatment. There were seven drug–drug pairs with a strong R^2^ value of above 0.7 in the linear regression analysis, including atezolizumab–apixaban, osimertinib–apixaban, erlotinib–apixaban, docetaxel–verapamil, pembrolizumab–apixaban, docetaxel–diltiazem and paclitaxel–apixaban. This indicated the possible association of atrial fibrillation with atezolizumab, osimertinib, erlotinib, pembrolizumab and paclitaxel; and hypertension with docetaxel. While our study suggests potential association between certain drug–drug pairs, these associations must be further investigated through more detailed clinical studies or patient-level real-world data that can account for specific circumstances under which these drugs are co-administered.

## Figures and Tables

**Table 2:** A heat map showing the correlation coefficient between 20 shortlisted cardiology drugs and 20 shortlisted cancer treatments (*** *p* < 0.001, ** *p* < 0.01, * *p* < 0.05).

	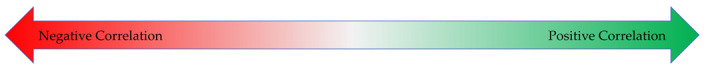																	
	Alteplase	Amiodarone	Amlodipine	Apixaban	Atenolol	Bisoprolol	Candesartan	Diltiazem	Doxazosin	Epinephrine	Lidocaine	Lisinopril	Losartan	Ramipril	Rivaroxaban	Streptokinase	Tenecteplase	Verapamil
Afatinib	−0.061	0.206	−0.372 **	−0.565 ***	0.354 **	−0.195	−0.357 **	0.279 *	−0.331 **	−0.407 ***	0.271 *	0.147	−0.322 **	−0.146	−0.341 **	0.233	0.308 *	0.345 **
Atezolizumab	0.495 ***	−0.380 **	0.545 ***	0.955 ***	−0.781 ***	0.312 *	0.500 ***	−0.435 ***	0.594 ***	0.242	−0.185	−0.430 ***	0.339 **	0.062	0.425 ***	−0.247 *	−0.222	−0.596 ***
Bevacizumab	0.329 **	0.039	0.692 ***	0.773 ***	−0.362 **	0.561 ***	0.611 ***	−0.082	0.674 ***	0.524 ***	−0.192	0.027	0.572 ***	0.423 ***	0.588 ***	−0.076	−0.253 *	−0.217
Carboplatin	0.275 *	0.366 **	0.775 ***	0.549 ***	0.004	0.725 ***	0.713 ***	0.311 *	0.753 ***	0.530 ***	0.196	0.400 **	0.721 ***	0.645 ***	0.820 ***	0.056	−0.136	0.200
Cisplatin	−0.295 *	0.648 ***	0.175	−0.394 **	0.741 ***	0.326 **	0.156	0.713 ***	0.129	0.256 *	0.237	0.747 ***	0.321 **	0.509 ***	0.299 *	0.248 *	0.050	0.657 ***
Cyclophosphamide	−0.034	0.623 ***	0.483 ***	0.026	0.416 ***	0.580 ***	0.450 ***	0.533 ***	0.481 ***	0.320 **	0.107	0.600 ***	0.571 ***	0.631 ***	0.488 ***	0.224	0.071	0.483 ***
Docetaxel	−0.393 **	0.783 ***	0.172	−0.425 ***	0.786 ***	0.450 ***	0.226	0.876 ***	0.158	0.159	0.196	0.806 ***	0.351 **	0.569 ***	0.282 *	0.451 ***	0.130	0.883 ***
Doxorubicin	0.359 **	0.330 **	0.583 ***	0.334 **	0.091	0.460 ***	0.436 ***	0.084	0.490 ***	0.380 **	0.189	0.305 *	0.541 ***	0.466 ***	0.517 ***	−0.133	0.023	0.093
Epirubicin	−0.346 **	0.769 ***	0.211	−0.411 ***	0.785 ***	0.418 ***	0.209	0.767 ***	0.136	0.327 **	0.153	0.809 ***	0.354 **	0.616 ***	0.369 **	0.383 **	0.152	0.836 ***
Erlotinib	−0.449 ***	0.497 ***	−0.425 ***	−0.896 ***	0.808 ***	−0.179	−0.394 **	0.537 ***	−0.466 ***	−0.195	0.209	0.521 ***	−0.228	0.050	−0.310 *	0.297 *	0.273 *	0.674 ***
Gefitinib	0.551 ***	−0.423 ***	−0.091	0.193	−0.409 ***	−0.257 *	−0.233	−0.627 ***	−0.098	−0.162	0.003	−0.447 ***	−0.249 *	−0.329 **	−0.240	−0.377 **	0.179	−0.491 ***
Gemcitabine	0.288 *	−0.149	0.493 ***	0.609 ***	−0.387 **	0.296 *	0.410 ***	−0.273 *	0.477 ***	0.247 *	−0.011	−0.111	0.371 **	0.219	0.424 ***	−0.246	−0.189	−0.366 **
Methotrexate	0.039	0.175	0.085	−0.123	0.332 **	0.132	0.077	0.132	0.031	0.013	0.041	0.321 **	0.138	0.205	0.145	0.003	0.061	0.281 *
Nintedanib	0.434 ***	−0.268 *	0.512 ***	0.818 ***	−0.641 ***	0.330**	0.451 ***	−0.327 **	0.548 ***	0.198	−0.210	−0.332 **	0.355 **	0.094	0.338 **	−0.245	−0.191	−0.478 ***
Osimertinib	0.407 ***	−0.298 *	0.572 ***	0.906 ***	−0.666 ***	0.340 **	0.510 ***	−0.408 ***	0.575 ***	0.386 **	−0.256 *	−0.321 **	0.398 **	0.145	0.447 ***	−0.235	−0.248 *	−0.536
Paclitaxel	0.466 ***	−0.242	0.584 ***	0.843 ***	−0.570 ***	0.397 **	0.514 ***	−0.270 *	0.596 ***	0.346 **	−0.125	−0.233	0.390 **	0.195	0.455 ***	−0.187	−0.229	−0.471 ***
Pembrolizumab	0.500 ***	−0.433 ***	0.454 ***	0.879 ***	−0.759 ***	0.224	0.380 **	−0.471 ***	0.494 ***	0.123	−0.206	−0.485 ***	0.217	−0.026	0.320 *	−0.290 *	−0.194	−0.635 ***
Pemetrexed	−0.141	0.181	0.128	0.011	0.082	0.133	0.091	0.061	0.069	0.224	−0.153	0.163	0.127	0.233	0.131	−0.007	−0.129	0.106
Vincristine	0.499 ***	−0.468 ***	0.332 **	0.744 ***	−0.708 ***	0.106	0.193	−0.541 ***	0.342 **	0.151	−0.159	−0.488 ***	0.090	−0.091	0.205	−0.372 **	−0.195	−0.607 ***
Vinorelbine	−0.452 ***	0.760 ***	0.189	−0.409 ***	0.779 ***	0.401 **	0.241	0.825 ***	0.156	0.177	0.175	0.780 ***	0.383 **	0.545 ***	0.310 *	0.358 **	0.075	0.786 ***

**Table 3:** Correlation between each shortlisted oncology drug and its most associated cardiology medication.

Oncology Drug	Cardiology Drug That Was Most Associated with Each Oncology Drug	Cardiovascular Disease(s) That the Corresponding Cardiology Drug Was Assumed to Treat
Afatinib	Atenolol	Ischaemia/hypertension
Atezolizumab	Apixaban	Atrial fibrillation
Bevacizumab	Apixaban	Atrial fibrillation
Carboplatin	Rivaroxaban	Arterial/venous thromboembolic event
Cisplatin	Lisinopril	Hypertension/cardiac failure/arrest
Cyclophosphamide	Ramipril	Hypertension/cardiac failure/arrest
Docetaxel	Verapamil	Arrhythmia/hypertension
Doxorubicin	Amlodipine	Hypertension
Epirubicin	Verapamil	Arrhythmia/hypertension
Erlotinib	Atenolol	Ischaemia/hypertension
Gefitinib	Alteplase	Myocardial infarction
Gemcitabine	Apixaban	Atrial fibrillation
Methotrexate	Atenolol	Ischaemia/hypertension
Nintedanib	Apixaban	Atrial fibrillation
Osimertinib	Apixaban	Atrial fibrillation
Paclitaxel	Apixaban	Atrial fibrillation
Pembrolizumab	Apixaban	Atrial fibrillation
Pemetrexed	Ramipril	Hypertension/cardiac failure/arrest
Vincristine	Apixaban	Atrial fibrillation
Vinorelbine	Diltiazem	Hypertension

**Table 4:** Drug–drug pairs which demonstrated a moderate or strong relationship (R^2^ ≥ 0.5).

Drug–Drug Pair		Linear Regression Analysis		
NSCLC Drug	Cardiotoxicity Drug	Coefficient	Intercept	R^2^ Value
Atezolizumab	Apixaban	1.337	4949.882	0.911
Osimertinib	Apixaban	1.204	5265.088	0.820
Erlotinib	Apixaban	−4.095	9610.698	0.802
Docetaxel	Verapamil	5.540	1848.673	0.779
Pembrolizumab	Apixaban	2.595	3926.500	0.773
Docetaxel	Diltiazem	1.661	−215.759	0.767
Paclitaxel	Apixaban	0.002	4595.091	0.711
Epirubicin	Verapamil	8.916	1077.783	0.699
Vinorelbine	Diltiazem	7.615	−248.661	0.681
Carboplatin	Rivaroxaban	82.480	221,044.146	0.672
Nintedanib	Apixaban	0.316	1242.011	0.669
Epirubicin	Lisinopril	171.813	100,349.747	0.655
Erlotinib	Atenolol	3.340	5815.185	0.653
Docetaxel	Lisinopril	100.603	120,115.002	0.649
Vinorelbine	Verapamil	23.997	1988.937	0.618
Docetaxel	Atenolol	7.436	2241.347	0.618
Epirubicin	Atenolol	12.625	823.243	0.616
Docetaxel	Amiodarone	19.449	19,261.962	0.614
Atezolizumab	Atenolol	−0.990	9483.021	0.610
Vinorelbine	Lisinopril	473.929	115,861.880	0.609
Vinorelbine	Atenolol	35.861	1778.631	0.607
Carboplatin	Amlodipine	0.908	2562.594	0.600
Bevacizumab	Apixaban	5.869	1829.886	0.597
Epirubicin	Amiodarone	32.446	15,888.632	0.591
Epirubicin	Diltiazem	2.474	−330.944	0.589
Vinorelbine	Amiodarone	91.768	18,413.891	0.577
Pembrolizumab	Atenolol	−2.024	10,352.082	0.575
Carboplatin	Doxazosin	0.168	483.829	0.567
Cisplatin	Lisinopril	109.250	140,300.077	0.558
Vincristine	Apixaban	170.599	4044.368	0.553
Cisplatin	Atenolol	8.214	3656.904	0.549
Carboplatin	Bisoprolol	0.814	2592.982	0.525
Carboplatin	Losartan	1.534	6331.187	0.519
Carboplatin	Candesartan	0.199	797.088	0.508
Cisplatin	Diltiazem	1.583	238.578	0.508
Vincristine	Atenolol	−146.874	10,476.737	0.501

## Data Availability

The data presented in this study are available on request from the corresponding author.

## Supplementary Materials

The following supporting information can be downloaded at: www.mdpi.com/article/10.3390/cancers17020311/s1, Table S1. A heat map showing the correlation coefficient between chemotherapies (*** *p* < 0.001, ** *p* < 0.01, * *p* < 0.05). Table S2. A heat map showing the correlation coefficient between target therapies (*** *p* < 0.001, ** *p* < 0.01, * *p* < 0.05). Table S3. A heat map showing the correlation coefficient between drugs used to treat cardiac failure (*** *p* < 0.001, ** *p* < 0.01, * *p* < 0.05). Table S4. A heat map showing the correlation coefficient between drugs used to treat myocardial infarction (*** *p* < 0.001, ** *p* < 0.01, * *p* < 0.05). Table S5. A heat map showing the correlation coefficient between drugs used to treat hypertension (*** *p* < 0.001, ** *p* < 0.01, * *p* < 0.05).
