# Supplementary material for: Cancer Therapy-Induced Cardiotoxicity: Results of the Analysis of the UK DEFINE Database
Source: Cancers (Basel). 2025 Jan 19;17(2):311. doi: 10.3390/cancers17020311 (PMC11763784; doi:10.3390/cancers17020311)
Supplement: Supplementary file 1 [file cancers-17-00311-s001.zip › cancers-3336292-supplementary.pdf]

Table S1. A heat map showing the correlation coefficient between chemotherapies (*** p < 0.001, ** p < 0.01, * p < 0.05).

Drug	Carboplatin	Cisplatin	Cyclophosphamide	Docetaxel	Doxorubicin	Epirubicin	Gemcitabine	Methotrexate	Paclitaxel	Pemetrexed	Vincristine	Vinorelbine
**Carboplatin**		0.306 *	0.484 ***	0.231	0.612 ***	0.340 **	0.369 **	0.042	0.515 ***	0.300 *	0.235	0.238
**Cisplatin**	0.306 *		0.527 ***	0.720 ***	0.140	0.740 ***	-0.203	0.191	-0.117	0.099	-0.504 ***	0.722 ***
**Cyclophosphamide**	0.484 ***	0.527 ***		0.489 ***	0.448 ***	0.614 ***	0.180	0.197	0.156	0.142	-0.088	0.559 ***
**Docetaxel**	0.231	0.720 ***	0.489 ***		0.061	0.847 ***	-0.483 ***	0.164	-0.404 ***	0.098	-0.665 ***	0.844 ***
**Doxorubicin**	0.612 ***	0.140	0.448 ***	0.061		0.204	0.386 **	0.125	0.234	-0.006	0.231	0.199
**Epirubicin**	0.340 **	0.740 ***	0.614 ***	0.847 ***	0.204		-0.272 *	0.227	-0.334 **	0.352 **	-0.530 ***	0.782 ***
**Gemcitabine**	0.369 **	-0.203	0.180	-0.483 ***	0.386 **	-0.272 *		-0.016	0.553 ***	0.037	0.577 ***	-0.212
**Methotrexate**	0.042	0.191	0.197	0.164	0.125	0.227	-0.016		-0.136	-0.046	-0.047	0.091
**Paclitaxel**	0.515 ***	-0.117	0.156	-0.404 ***	0.234	-0.334 **	0.553 ***	-0.136		0.110	0.654 ***	-0.381 **
**Pemetrexed**	0.300 *	0.099	0.142	0.098	-0.006	0.352 **	0.037	-0.046	0.110		0.044	-0.030
**Vincristine**	0.235	-0.504 ***	-0.088	-0.665 ***	0.231	-0.530 ***	0.577 ***	-0.047	0.654 ***	0.044		-0.708 ***
**Vinorelbine**	0.238	0.722 ***	0.559 ***	0.844 ***	0.199	0.782 ***	-0.212	0.091	-0.381 **	-0.030	-0.708 ***	

Table S2. A heat map showing the correlation coefficient between target therapies (*** p < 0.001, ** p < 0.01, * p < 0.05).

Drug	Afatinib	Bevacizumab	Erlotinib	Gefitinib	Nintedanib	Osimertinib
**Afatinib**		-0.695 ***	0.738 ***	0.061	-0.440 ***	-0.835 ***
**Bevacizumab**	-0.695 ***		-0.673 ***	-0.068	0.677 ***	0.830 ***
**Erlotinib**	0.738 ***	-0.673 ***		-0.268 *	-0.757 ***	-0.940 ***
**Gefitinib**	0.061	-0.068	-0.268 *		0.293 *	0.140
**Nintedanib**	-0.440 ***	0.677 ***	-0.757 ***	0.293 *		0.774 ***
**Osimertinib**	-0.835 ***	0.830 ***	-0.940 ***	0.140	0.774 ***	

Immunotherapy: Atezolizumab and Pembrolizumab → Correlation Coefficient: 0.907 ***.

Table S3. A heat map showing the correlation coefficient between drugs used to treat cardiac failure (*** p < 0.001, ** p < 0.01, * p < 0.05).

Drug	Epinephrine	Lisinopril	Losartan	Ramipril
Epinephrine		0.331 **	0.453 ***	0.458 ***
Lisinopril	0.331 **		0.635 ***	0.807 ***
Losartan	0.453 ***	0.635 ***		0.893 ***
Ramipril	0.458 ***	0.807 ***	0.893 ***	

Table S4. A heat map showing the correlation coefficient between drugs used to treat myocardial infarction (*** p < 0.001, ** p < 0.01, * p < 0.05).

Drug	Alteplase	Streptokinase	Tenecteplase
Alteplase		-0.163	0.292 *
Streptokinase	-0.163		0.017
Tenecteplase	0.292 *	0.017	

Table S5. A heat map showing the correlation coefficient between drugs used to treat hypertension (*** p < 0.001, ** p < 0.01, * p < 0.05).

Drug	Amlodipine	Atenolol	Bisoprolol	Candesartan	Diltiazem	Doxazosin	Lisinopril	Losartan	Ramipril	Verapamil
Amlodipine		0.012	0.909 ***	0.894 ***	0.318 *	0.941 ***	0.454 ***	0.894 ***	0.827 ***	0.205
Atenolol	0.012		0.211	0.005	0.684 ***	-0.078	0.826 ***	0.243	0.446 ***	0.804 ***
Bisoprolol	0.909 ***	0.211		0.915 ***	0.596 ***	0.899 ***	0.651 ***	0.916 ***	0.942 ***	0.481 ***
Candesartan	0.894 ***	0.005	0.915 ***		0.423 ***	0.902 ***	0.456 ***	0.903 ***	0.824 ***	0.277 *
Diltiazem	0.318 *	0.684 ***	0.596 ***	0.423 ***		0.339 **	0.822 ***	0.505 ***	0.692 ***	0.867 ***
Doxazosin	0.941 ***	-0.078	0.899 ***	0.902 ***	0.339 **		0.390 **	0.881 ***	0.787 ***	0.192
Lisinopril	0.454 ***	0.826 ***	0.651 ***	0.456 ***	0.822 ***	0.390 **		0.635 ***	0.807 ***	0.833 ***
Losartan	0.894 ***	0.243	0.916 ***	0.903 ***	0.505 ***	0.881 ***	0.635 ***		0.893 ***	0.412 ***
Ramipril	0.827 ***	0.446 ***	0.942 ***	0.824 ***	0.692 ***	0.787 ***	0.807 ***	0.893 ***		0.623 ***
Verapamil	0.205	0.804 ***	0.481 ***	0.277 *	0.867 ***	0.192	0.833 ***	0.412 ***	0.623 ***	

Arrhythmia: Amiodarone and Verapamil → Correlation Coefficient: 0.796 ***.

Arterial / Venous Thromboembolic Event: Rivaroxaban and Streptokinase → Correlation Coefficient: 0.149.

Ischaemia: Atenolol and Bisoprolol → Correlation Coefficient: 0.211.

Atrial Fibrillation: Apixaban only.

Tachycardia: Lidocaine only.
